# Current Applications of Bionanocomposites in Food Processing and Packaging

**DOI:** 10.3390/polym15102336

**Published:** 2023-05-17

**Authors:** João Ricardo Afonso Pires, Carolina Rodrigues, Isabel Coelhoso, Ana Luisa Fernando, Victor Gomes Lauriano Souza

**Affiliations:** 1 MEtRiCS, CubicB, Departamento de Química, NOVA School of Science and Technology (FCT NOVA), Campus de Caparica, Universidade Nova de Lisboa, 2829-516 Caparica, Portugal; jr.pires@campus.fct.unl.pt (J.R.A.P.); cpe.rodrigues@campus.fct.unl.pt (C.R.); 2 LAQV-REQUIMTE, Departamento de Química, NOVA School of Science and Technology (FCT NOVA), Campus de Caparica, Universidade Nova de Lisboa, 2829-516 Caparica, Portugal; imrc@fct.unl.pt; 3 INL—International Iberian Nanotechnology Laboratory, Av. Mestre José Veiga s/n, 4715-330 Braga, Portugal

**Keywords:** nanotechnology, food safety, food technology, encapsulation, active packaging, intelligent packaging, biodegradable polymers

## Abstract

Nanotechnology advances are rapidly spreading through the food science field; however, their major application has been focused on the development of novel packaging materials reinforced with nanoparticles. Bionanocomposites are formed with a bio-based polymeric material incorporated with components at a nanoscale size. These bionanocomposites can also be applied to preparing an encapsulation system aimed at the controlled release of active compounds, which is more related to the development of novel ingredients in the food science and technology field. The fast development of this knowledge is driven by consumer demand for more natural and environmentally friendly products, which explains the preference for biodegradable materials and additives obtained from natural sources. In this review, the latest developments of bionanocomposites for food processing (encapsulation technology) and food packaging applications are gathered.

## 1. Introduction

Packaging plays a key role in the food industry thanks to its multiple functions. It represents a coordinated system for food transportation, distribution, storage, and end use, providing the means to ensure that the product reaches the final consumer under optimal conditions. The packaging serves as a container for the product, protecting it from shocks, vibrations, and compressions, and it also plays a vital role in the preservation of food, acting as a barrier to physical, chemical, and biological (re)contamination. Overall, it contributes to increasing product shelf life throughout the distribution and storage chain, maintaining its quality and safety, and reducing food waste [1].

Food processing is related to and interlinked with food packaging. Food processing can be defined as the systematic processes used in the food industries to physically, biologically, and chemically change fresh food. These processes contribute to improving food safety and extending its shelf-life, by eliminating or reducing spoilage and pathogenic microorganisms, enzymatic activity, and biochemical changes. Besides contributing to food preservation, it also contributes to improving the edibility, organoleptic quality, and sensorial quality of the food, easing distribution and marketing.

In recent years, the development of novel food packaging (modified atmosphere packaging (MAP) and active/intelligent packaging) and novel food preservation techniques (e.g., encapsulation of additives) have not only contributed to enhancing food’s shelf life but also its safety and quality—therefore bringing convenience to consumers [2,3]. Indeed, the search for new, better, and more sustainable food preservation techniques and food packaging options is an ongoing process that accompanies the success of the world’s largest food industries. The fulfillment of this stimulus is associated with more substantial profits, and with more satisfied consumers who desire nutritious nourishments with less pathogen contamination and few changes in organoleptic properties [4]. Currently, nanotechnology represents the most dominant driver for transporting traditional industries into a modern generation. The food industry is following this increasing tendency, and as such, novel ideas and opportunities have recently emerged for reshaping the way that food is processed and packed [5,6]. Bionanocomposites are hybrid materials, comprised of bio-based polymers and inorganic/organic nanoparticles (NPs), i.e., particles exhibiting at least one dimension on the nanometer scale [7]. In the dynamic process of the search for new and more sustainable materials for food processing and food packaging, biodegradable and renewable materials represent a viable and important choice [8,9]. Moreover, these materials fill the consumers’ preference for more natural and sustainable products, favoring materials that cause less environmental impact, and favoring food additives from natural sources over synthetic ones. Therefore, this manuscript presents a literature review of recent information on the use of bionanocomposites as carriers of active compounds and also as food packaging materials. This literature review targets the types of nanomaterials most used/studied, the preparation techniques used to encapsulate bioactive compounds, and the techniques used to incorporate NPs into food packaging materials. Moreover, some examples of their applications, which are more focused on food packaging, are also presented with the main results. This range of nanomaterials is undergoing intensive development, and the paper focuses on the most recent and innovative technologies that are being developed in this field. Gathering the current knowledge in this area in the same manuscript will allow stakeholders to better understand the alternative options for protecting food products, and establish which are more effective and less burdensome to the environment.

## 2. Bionanocomposites Preparation for Food Processing Applications

Bioactive compounds, such as phenolic compounds, essential oils, carotenoids, vitamins, probiotics, peptides, and polyunsaturated fats (PUFs) are an asset when we talk about food, as they confer a broad variety of functional properties, such as antimicrobial, antioxidant, anti-inflammatory, nutraceutical/nutritional or even anticarcinogenic activity [10,11,12,13]. However, the direct incorporation of pure biocompounds or extracts in food remains an arduous task due to their chemical instability, easy oxidation under specific conditions, fast release, hydrophobicity, and low solubility that often leads to poor bioavailability. Additionally, the released amount must be restricted, to avoid the risk of causing acute toxicological problems to the consumer [14,15,16]. Therefore, novel technologies that ensure the controlled release and stability of such biomolecules in food are essential.

Nanotechnology through encapsulation with nanocarriers can represent a promising approach to increasing the solubility, stability, and bioavailability of bioactive compounds. Encapsulation is understood as the technique that allows the coating of active agents designated as nuclei or core, with an external transport material commonly called a membrane, capsule, shell, or wrapper. The encapsulation technique is already frequently used by the food industry to preserve organic particles responsible for imparting aroma and flavor once these molecules typically sustain lower molecular weights and can easily volatilize during food processing/manufacturing and storage [17]. The bioactive components that are meant to be protected are coated with a polymer that encapsulates the active components and preserves them from the action of oxygen, moisture, heat, pH changes, and light, which prolongs their stability and functionality [18]. Nanocomposites, by assuming a position of nano-scale delivery system, help to prevent the loss of functionality, forming protective barriers that secure the active substances from degrading and guaranteeing a controlled release on specific targets [15,19]. However, different polymers behave differently as protective barriers. Polysaccharides, for example, are an effective barrier to gases, such as O_2_ and CO_2_, and prevent oxidation due to the well-ordered hydrogen-bonded network configuration. Reinforcement of biobased polymers with nanoparticles alters the diffusion path of different molecules and can contribute to improving their barrier properties. Despite this, the hydrophilic nature of some of the polymers (e.g., chitosan) results in poor water vapor and moisture barrier properties [20]. Encapsulation also reduces the impact of those bioactive compounds on the product’s sensory properties and limits their interaction with other food ingredients [21].

Countless food-grade biopolymers (e.g., natural or modified polysaccharides, gums, proteins, lipids, and synthetic polymers) can be used as nano-encapsulating agents; however, before their use, the choice of a nanocarrier should respect tight evaluation criteria. Since the key objective of these capsules/nanocapsules is to be incorporated into food matrices, it is essential to ensure consumer health security, and consequently, the capsules/nanocapsules must be considered as GRAS (generally recognized as safe). Thus, most of the wall materials used are food base compounds (polysaccharides, proteins, and lipids). Accordingly, the impact of nanoparticles on human health and the environment should be investigated in-depth, and accompanied by the creation of regulatory criteria [22,23,24,25]. Moreover, each bioactive compound exhibits distinct functional properties, which demands extensive laboratory study to pursue the best coating substance to ensure maximum compatibility without chemical reactions between the compounds, to keep the core stable. Indeed, the physicochemical and rheological behaviors presented by the capsule material determine its capacity to correctly fill this purpose. These capsule materials should present fine emulsifier properties, low viscosity at high concentrations, good dissolution, and network-forming characteristics [19]. Nevertheless, the success of the biomolecule’s encapsulation is not solely dependent on the correct synergy achieved after the contact between the core and the membrane; it is also dependent on the technique applied to carry out this methodology and the ultimate purpose of the product. When observing the present-day literature, it can be seen that a wide variety of techniques have been proposed for the encapsulation of bioactive compounds. The most frequently used encapsulation techniques are emulsion-based, extrusion-dripping, coacervation, spray-drying, spray-chilling, freeze-drying, and fluidized-bed-coating [26]. However, the choice of method is highly dependent on the type and characteristics of the core and the coating material, and none of the proposed systems can be officially recognized as the standard system [19].

Nanoencapsulation is a complicated topic due to the countless possible combinations of biopolymers, bioactive compounds, and encapsulation techniques involved to produce it. It can be challenging to accurately summarize this information in one manuscript. Therefore, this review aims to simplify the information by focusing on some established biopolymer-based nanoencapsulation methods, such as emulsification, extrusion, and drying techniques, electrohydrodynamic processes, cryogelation, and complex coacervation, which can be used to create different nanostructures for hydrophobic food bioactive compounds, including nanoemulsions, nano hydrogels, nanofibers, nanospheres, nanoliposomes, and nanosponges [27,28,29]. Table 1 has been provided to help organize this information.

### 2.1. Emulsification Techniques

The most common method employed in encapsulation is emulsification, which represents the mixing of two immiscible substances (e.g., emulsified oil and an aqueous system) using a stabilizing agent (emulsifier) to create one stable phase, i.e., an emulsion. Nanoemulsion-based encapsulation consists of a colloidal dispersion composed of three phases: a dispersed/discontinuous internal phase, a continuous external phase, and an interphase called an emulsifier. The emulsifier contains essential low molecular-weight surfactants to decrease the tension and interfacial energy, allowing a stable mixture between the oily and aqueous phases [50,51]. Nanoemulsions have certain advantages over their macro-sized counterparts, primarily due to their incredibly small size. These advantages include enhanced stability, improved optical properties, and better-releasing control mechanisms. These nanometer-sized ultrafine emulsions can now be obtained by processes involving high energies, such as high-pressure valve homogenizers, microfluidizers, and sonication, or less energetic approaches, such as phase inversion or spontaneous emulsification [17].

Membrane emulsification can also be used to obtain nanoemulsions [52]. This method involves the use of applied pressure to force the dispersed phase to permeate through a membrane into the continuous phase. The resulting droplet size is primarily controlled by the choice of the membrane pore and not by the generation of turbulent droplet break-up [52]. The apparent shear stress is lower than the emulsification systems mentioned above because small droplets are directly formed by permeation of the dispersed phase through the pores, instead of by the disruption of large droplets in zones of high energy density. Outside the possibility of using shear-sensitive ingredients, emulsions with narrow droplet size distributions can be produced.

Thus, membrane emulsification offers compact devices for the preparation of nanoemulsions with low energy consumption, tunable droplet size, monomodal distribution, and high encapsulation efficiency of entrapped functional components without shear or thermal degradation. Membranes for micro/nano-emulsification should have narrow pore size distribution, low resistance to flow, excellent mechanical and chemical durability, high thermal resistance, and wettability that can easily be modified, and they should be biocompatible, sterile, and cheap [53,54,55].

Both techniques have their advantages and disadvantages. When energy is lower, the associated costs will also decrease; on the other hand, the use of surfactants will increase. When the process exploits high energies, the control of the size of the droplets is much more precise, but as a result, there is a rise in costs. To improve thermal stability and thus upgrade industrial applicability, nanoemulsions are frequently dried [50].

### 2.2. Drying Techniques

Freeze-drying and spray-drying are the most widely used encapsulation drying techniques in the food industry. These can be implemented as the last step of the nanoencapsulation process. Spray-drying is an economical and prompt method whereby an aqueous solution or emulsion is atomized against a hot chamber, forming spherical particles of exact microscopic size containing the intended biomolecules, which are later recovered with the help of a cyclone [29,56]. This method is already conventionally implemented in the elaboration of dried food, food additives, and flavor. However, poor yield, low control of particle size, and the need to use high temperatures represent obstacles to the encapsulation of volatile or thermosensitive compounds, which highlights the need to optimize this technique [19,28]. Moreover, in conventional spray-drying techniques, the cyclone separating units cannot collect very small particles, which limits their use to produce nanoparticles. Recently, a new spray-dryer (nano spray-dryer), produced by BÜCHI Labortechnik AG [33] and equipped with an electrostatic separation unit, has been designed successfully to address this issue, enabling the production of dried powder containing nanoparticles with a uniform size distribution [57]. Indeed, several works have been published recently with different nanoparticle production of encapsulated active compounds using this novel technology [33,34,35,36].

Freeze-drying is a phased and more effective method for producing stable nanoparticles. This method is more suitable for encapsulating molecules that are sensitive to thermal degradation as it is processed under a low temperature and pressure. The water present in the droplets is removed in four steps: freezing, sublimation, desorption, and storage. Despite offering the ampler capacity to retain volatile compounds, colors, aromas, and flavors, the high cost and drying time are barriers to industrial applicability [17,19,58].

By combining the advantages of freeze-drying and spray-drying, a new technique called spray-freeze-drying has been refined. As the name of the technique implies, this is a blend of the previous techniques that is characterized by a set of steps, beginning with droplet formation and freezing, and ending in their sublimation. As happens with spray-drying, this process begins with the atomization of the aqueous solution or emulsion in nano-sized particles or droplets. The difference lies in the fact that they are scattered against a fluid at very low temperature and pressure values, leading to its cooling and sublimation [59]. This approach is still at the optimization stage, demanding the perfect adaption of the two techniques that support it. However, as it operates at very low pressures and temperatures, the costs are elevated, making it difficult to up-scale from the laboratory to the industrial level. The handling of cryogenic fluids such as liquid nitrogen for the cooling of the droplets must also be precise and careful, which constitutes an obstacle. However, it is a technique with very good future potential for application to nanoencapsulation [17,60].

Elik et al. (2021) [61] performed a comparative study of two different techniques (spray-freeze-drying and nano spray-drying) to produce nanoparticles containing encapsulated carotenoid enriched-flaxseed oil or only flaxseed oil in maltodextrin/pectin + wax. The nano spray-dryer technique resulted in superior encapsulation efficiency but inferior flow properties. In terms of the oxidation stability of the nanocapsules, the spray-freeze-drying technique provided the production of encapsulated oils with lower rancidity and a longer shelf-life during storage in comparison to the nano spray-dryer. Moreover, the inclusion of carotenoids into the system also contributed to the retardation of the lipid oxidation of the capsules. The authors demonstrated that the methods were suitable for the encapsulation of oils that are sensitive to oxidation [61].

### 2.3. Extrusion Techniques

Extrusion techniques represent another attractive strategy, whereby the active substance is embedded into the biopolymer nanocarrier solution, usually polysaccharides, enabling an effective encapsulation of an ample range of hydrophilic or hydrophobic active compounds. In this laboratory-scale practice, the solution is loaded inside a syringe and posteriorly forced to pass through a nano-scale needle into a gelling medium [29,62]. These techniques are considered advantageous on account of operative mild conditions in terms of temperature or solvents. Moreover, it is possible to operate an extruder with regulated temperatures and speeds. It is also possible to accurately adapt the needles to dimensions on the nanometer scale, as this technique typically produces droplets with particle sizes around the microns. On the other hand, the excessive porosity of the particles endures a challenge to overcome, as it hinders a controlled release of the active compounds and their stability. That said, this technique is limited to membrane materials and is hard to scale up. Specific strategies can be contemplated to upgrade the extrusion technique, such as the application of a multi-nozzle system or rotating disks, centrifugal extrusion, co-extrusion, or melt extrusion [19,63].

### 2.4. Electrohydrodynamic Process

Electrohydrodynamic processes that denominate electro-spinning and electro-spraying are advantageous novel approaches for nanoencapsulation, and allow the preparation of eco-friendly, active food electrospun materials using biopolymers and their bionanocomposites. This technology is highlighted due to its capacity to be performed at ambient temperatures at low cost and scalability when compared to other techniques, which is singularly appropriate for sensitive and volatile compound encapsulation structures [17,27,28]. The polymer concentration sets the difference between the two techniques, while in electro-spinning stabilized nanofibers are produced with high-concentration polymers, in electro-spraying fine droplets or particles are produced with low-concentration polymers [17]. The principle behind the two methodologies is derived from the use of high voltage potential electric fields for spraying the polymer/bioactive compound solution or emulsion to create the nanofibers (electro-spinning) or particles (electro-spraying) [28]. The ability to carry out this process at low temperatures ensures the stability of the active substances. In addition, the high surface-to-volume ratio and the nanometric size of the fiber/droplet facilitate a quick and effective release of the biomolecules, preventing contamination by microorganisms. Hence, the prospects and ambitions for this field in the future are huge and it is essential to produce more research to better understand the particle size and morphology of the nanoparticles, and conduct additional tests to enlarge the range of biopolymers and active compounds that can be employed in this technique [28,64].

### 2.5. Cryogelation

Cryogelation is a recognized method intended to produce hydrophilic nanogels, also called nano hydrogels, hydrophobic nanogels, or nano organogels [27]. It is a gelation process that takes place under semi-frozen conditions, resulting in a polymer network that is cross-linked around ice crystals [65]. This three-dimensional polymerization network shaping embraces three fundamental steps to form a crosslinked nanogel arrangement: (i) freezing, (ii) gel formation, and (iii) defrosting of the reagent/polymer solution. Nanogels have recently attracted scientific and industry attention because, as hydrogels, their well-defined structures can shift their morphology and shape when subjected to external inputs such as temperature, pH, salt, or magnetic fields. The faster swelling/deswelling characteristic feature of nanogels allied with their excellent permeation abilities, high load capacity, controlled release of active compounds, and faculty to enhance the solubilization of numerous hydrophobic substances, are important advantages when applied as nanocarriers [27,66,67,68].

### 2.6. Complex Coacervation

Complex coacervation is a simple and one of the most relevant nanoencapsulation methodologies, is based on the electrostatic interaction between two hydrophobic colloids with reverse charges, and is capable of formulating water- and heat-resistant nanomembranes [17,19]. The complex coacervates droplet shell originates after the application of heating, desolvation, or crosslinking techniques to the newly emulsified layer that covers the nucleus component developed during the mixing of three immiscible phases: bioactive core substance, biopolymer matrix, and continuous liquid phases [29,63]. This technique holds driver advantages, such as high loading and encapsulation efficiency, the use of mild condition and non-toxic solvents, and the possibility of forming a large number of complexation bonds, displaying why it is one most suitable methods for bioactive compounds [19,50]. It is also feasible to achieve nanocapsules with contrasting attributes and sizes by manipulating the temperature, type of biopolymer, ion concentration, and bioactive ingredient proportion [63]. Nonetheless, the particle dispersion typically requires a secondary post-treatment, for instance, spray-drying or freeze-drying, to acquire a more optimized nanomaterial [29].

There has been a recent trend of investing in the development of new nanometer-sized encapsulations that can safely and efficiently transport bioactive compounds to food. This review highlights the numerous possibilities for nanocarriers that can be found in the literature, depending on the coating material, core, and technique used to create them. Nanoencapsulation techniques reveal more significant encapsulation efficiency, increased stability, and a better handle on release profile compared to traditional encapsulation. Nonetheless, there is still a lot to be explored in this field, especially with research for novel upgraded cost-effective nanoencapsulation systems that can be upscaled efficiently at the industrial level. Another critical aspect that remains to be established is the regulation to assure the safety of these products. To this end, therefore, it is crucial to focus on the biological effects that nanoparticles can cause on the human body, carrying out toxicity, migration, absorption, and digestion tests.

## 3. Bionanocomposites and Nanoparticles for Food Packaging Applications

The scope of food packaging materials is not only to maintain food quality but also to guarantee food safety [69]. Petroleum-based plastic materials have been largely used in food packaging due to their excellent properties, quality, processability, and low cost. Plastic materials can be used in various forms of packaging, but the main problem is that they can cause huge environmental problems [70]. They are not biodegradable, and their waste disposal contaminates soil, water, and air [69]. Therefore, an alternative is needed, and recently, new packaging materials have been developed and can be found in the market. New packaging materials, such as bio-based polymers, carry different and new properties. Biopolymers are obtained from renewable sources and are considered biodegradable [8]. Moreover, through the incorporation of active compounds, some features can be added, such as antimicrobial and antioxidant properties [69,71].

**Figure 1:**
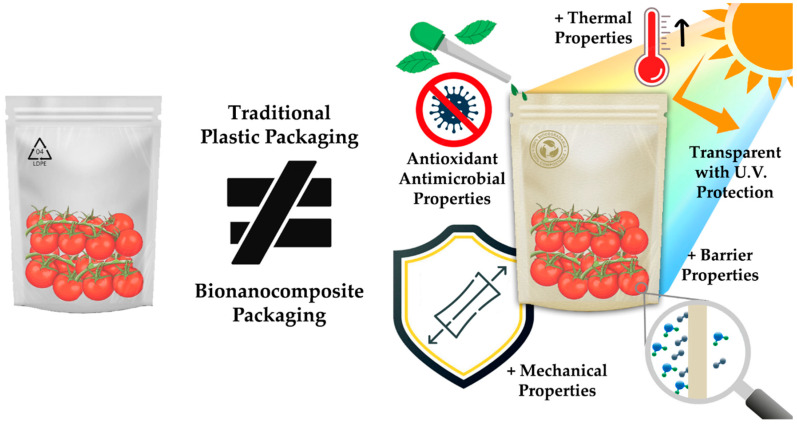
Advantages of bionanocomposites compared to traditional petroleum-based packaging.

Bionanocomposites belong to a class of composites that are composed of a bio-based polymeric matrix reinforced with fillers that have a dimension within the nanometer scale range of 1 to 100 nm. Additionally, other bioactive substances can be incorporated to confer active or intelligent properties to preserve or detect/indicate foodstuff alterations, respectively [20,72,73,74]. These novel materials are increasingly being used in food packaging to reduce food losses and improve safety, since they offer several advantages over traditional biopolymers, such as improved water vapor and gas barrier properties, and thermal stability, mechanical strength, UV barrier, biodegradability, and antimicrobial properties (as illustrated in Figure 1) [72,75,76,77].

### 3.1. Nanoparticles Used in Food Packaging

The reinforcing nanoparticles can be obtained from various materials, including polymers, metals, and metal oxides [9]. The primary motivation behind their addition is the high surface area per weight ratio attributed to them. This feature enhances the interaction between the nanoparticles and polymer chains, ultimately resulting in superior material properties and performance, for example, allowing greater interactions with gases and liquids, making nanocomposites excellent candidates for use as barrier materials [9,78,79]. Moreover, the bionanocomposites exhibit a range of properties that are dependent on various factors, such as the origin, size, shape, and surface chemistry of nanoparticles, as well as the polymer used [20].

Nanoparticles can improve the mechanical properties of bionanocomposites in different ways. Their high aspect ratio increases the interfacial area between the nanoparticles and matrix, leading to enhanced load transfer and stress distribution with the bionanocomposite, resulting in increased stiffness, strength, and toughness [80]. The surface modification of the nanoparticles, which introduces functional groups that promote chemical bonding, could be essential for improving the interfacial adhesion between components, resulting in increased mechanical properties. Moreover, the correct dispersion of nanoparticles within the matrix is crucial to the performance of the resulting material. When this condition is achieved, the nanoparticles form a homogenous network structure that can significantly enhance the material stiffness and strength, even with the incorporation of small amounts of nanoparticles [7,81].

Nanoparticles have also the potential to act as catalysts and promote thermal stability by lowering the activation energy required for thermal degradation, making them an ideal choice for high-temperature applications. For instance, metal oxide nanoparticles are capable of catalyzing the oxidation of volatile organic compounds, thereby enhancing the thermal stability of polymer matrices. By reducing the energy barrier for the degradation reactions, nanoparticles can effectively prevent or delay the breakdown of the polymer matrix, leading to improved performance and durability [82,83].

Overall, bionanocomposites demonstrate an enormous potential to transform the food packaging industry by providing a safer, more sustainable, and more efficient way to store and transport food products.

The most commonly used nanofillers are nanoclays, metal and metal oxide nanoparticles, nanocellulose, and carbon nanotubes, which are briefly described below.

#### 3.1.1. Nanoclays

Nanoclays are used as fillers to improve some properties of the polymer. The incorporation of nanoclays into the polymer matrix has been shown to be effective in the improvement of the physical, barrier, and mechanical properties of bionanocomposites [20,84,85,86].

Montmorillonite (MMT) is one of the most used and studied clays because of its low cost, high active surface, ability to improve mechanical strength and barrier properties when introduced to bionanocomposites [85]. Due to its polarity, MMT needs a modification process based on cation exchange, resulting in organo-modified layered silicates [87]. MMT is a hydrate alumina-silicate layered clay, and its negative charge is balanced with exchangeable cations; for example, Na^+^ and Ca^2+^ [73]. Halloysite (Hal) is another type of nanoclay that is used as reinforcement. Hal is a mineral natural aluminosilicate clay that has a hollow and tubular shape [88].

#### 3.1.2. Metal and Metal Oxide Nanoparticles

Inorganic nanoparticles have been used as reinforcement for bionanocomposites, and the most common are metal or metal oxide nanoparticles. Metal nanoparticles, such as silver (AgNPs), zinc (ZnNPs) and gold (AuNPs), are commonly used in food packaging [89,90,91,92]. The most used metal oxide nanoparticles are zinc oxide (ZnO NPs), titanium oxide (TiO_2_ NPs), and magnesium oxide (MgO NPs) [72]. When using metal or metal oxide nanoparticles, it is important to focus on two important aspects, control of the growth, size, and distribution of the matrix and NPs structure and their stabilization, to assure their function on the material [72]. Metal oxide nanoparticles can decrease the hydrophilicity of the biopolymer, as well as enhance the UV-light barrier and provide antimicrobial and antioxidant properties [91,93]. As an example, ZnO NPs are used to improve packaging material properties due to their high stability, photocatalytic activity, and antibacterial activity [94,95]. TiO_2_ NPs are used due to their ability to reduce water vapor permeability and form a barrier against moisture due to their low hydrophilicity [96]. On the other hand, biogenic AgNPs synthesized by the reduction of AgNO_3_ with the ethanolic extracts of *Eucaliptus camaldulensis* do not alter the properties of polyvinyl alcohol-chitosan films but rather increase their antimicrobial and antioxidant properties [91]. The functionalization of wastepaper with bioactive AgNPs, to produce reinforced recycled paper with antimicrobial properties against both Gram-positive and Gram-negative foodborne bacteria in a sustainable waste management approach, has also been prepared by Nwabor et al. (2021) [92].

The green synthesis method is a current trend to produce bioactive metal oxide nanoparticles, which have been applied in the development of active packaging with potential use in the extension of the shelf-life of food products [95,97,98,99].

#### 3.1.3. Nanocellulose

Nanocellulose is extracted from lignocellulosic matrices and when added to a polymeric matrix allows enhancement of the mechanical and gas barrier properties, especially to oxygen [100,101,102,103]. Once it is a renewable and recycled material, it can be used as a nanofiller in packaging products [10,72].

Three types of nanocellulose are commonly used for the reinforcement of bionanocomposites: nanocrystalline cellulose (NCC), nanofibrillated cellulose (NFC), and bacterial nanocellulose (BNC). The differences between them reside in their source and extraction methods, morphology, crystallinity degree, and particle size [104]. Nanocrystalline cellulose can be obtained through enzyme or acid hydrolysis from different cellulosic biomasses, producing a gel, liquid, or powder through the elimination of the amorphous areas [72]. Nanofibrillated cellulose is extracted with mechanical methods from cellulose fibers and is characterized by being long, flexible, and entangled [104]. Nanofibrillated cellulose is constituted by the crystalline and amorphous areas of cellulose after the removal of the lignin fraction [105]. Bacterial nanocellulose is generally extracted from Gram-negative bacterial cultures and when compared to nanocellulose extracted from biomass, the former pursues a high molecular weight and crystallinity [106].

#### 3.1.4. Carbon Nanotubes

Carbon nanotubes are organic nanofillers constituted by an ultrathin layer of carbon fibers [74]. The nanotubes can be divided into multi-walled carbon nanotubes and single-walled carbon nanotubes [89]. A cylinder with a diameter of 1 nm constitutes a single-walled carbon nanotube (SWCNT), while a multi-walled carbon nanotube (MWCNT) is composed of multiple concentric cylinders ranging in diameter from 2 to 100 nm and separated by a distance of 0.35 nm. These tubes can extend up to tens of microns in length [107]. The use of carbon nanotubes shows some constraints due to challenges in processing, dispersion, and high cost [108]. The use of carbon nanotubes in food packaging is linked with the improvement of antibacterial, thermal, and mechanical properties, with good processability and low weight [76,89,109].

### 3.2. Incorporation Techniques

To introduce nanofillers in the polymer matrix, different preparation methods are used, such as intercalation, in situ polymerization, and melt processing.

In situ polymerization occurs when the nanoparticle is mixed with the polymer solution [9]. The intercalation method is used when nanoparticle and polymer pellets are pressed to help their dispersion and exfoliation to form a polymer matrix [9]. Melt processing is used when the polymer is being processed at the same time, and to disperse the nanoparticle in the biopolymer, promoting the homogenization of the film-forming solution [87,110]. Compared to the other methods for nanocomposite incorporation, melt intercalation does not require the use of solvents, is a flexible method and reduces the interfacial tension of matrix-filler interaction [100].

To illustrate the variances in the production of bionanocomposites, a practical example can be provided based on the use of a biopolymer and nanoclays. Three configurations of the resulting nanocomposite are possible, namely: (1) intercalated (or partially exfoliated), (2) exfoliated, or (3) tactoid (micro-composite) [111,112]. The intercalated type is characterized by the polymer chains interacting with the clay interlayers and allowing the layers to remain stacked [69]. Thus, the nanoclays are considered to be partially exfoliated, and improvements in the barrier, mechanical and thermal properties are reported [113]. In the exfoliated type, there is a loss of the clay structure leading to the complete dispersion of the clay onto the polymer matrix, which, for example, creates a more tortuous path responsible for the enhancement of gas barrier properties. The achievement of an exfoliation configuration is very important and desired, as this results in the best improvement of the material’s functional properties compared to the other two types of configurations [76,114]. In the tactoid type, the expansion of the clay structure does not occur, and in this case, the nanocomposite is not formed but is only a micro-composite [69]. The tactoid configuration is, therefore, not desired when preparing a nanocomposite, as it does not reflect significant gains in the improvement of the material’s properties. The differences in the dispersion of the NPs into the polymeric chain are related to the failure to separate the silicate layers; thus, in tactoid or intercalated configurations, the energy necessary to successfully enhance the distances between the silicate layers is not achieved, and the use of a more robust dispersing method should be employed [73,115].

### 3.3. Examples of Bionanocomposites Application in Food Packaging

Several studies with different bionanocomposites have been conducted over the past few years concerning the development, evaluation, and application of food packaging. Some of these are reported and summarized in Table 2.

Alexandre et al. (2016) [116], studied the production of gelatin-based films with MMT reinforcement and a nanoemulsion ginger essential oil (GEO). The addition of MMT to the films coupled with GEO improved their thickness and diminished the water solubility and moisture content. MMT only also decreased the superficial hydrophobicity and increased the roughness of the films [116]. Souza et al., 2018 developed bionanocomposites of chitosan/MMT with the incorporation of rosemary essential oil (REO) to form a bionanocomposite film, and evaluated its activity in fresh poultry meat. The incorporation of MMT reduced film water vapor and oxygen permeability. On the other hand, MMT addition to the bionanocomposite led to a decrease in the bioactivity of the films and diminished the release of the bioactive compounds to poultry meat when compared to the films without MMT [117]. The use of cellulose nanocrystals (CNC) extracted from sugarcane bagasse as reinforcement in a polyvinyl alcohol/carboxymethyl cellulose (PVA/CMC) blend has been studied for food packaging applications [119]. The use of CNC increased the tensile modulus and strength of the bio-based films and did not affect the transparency level of the films. The water vapor permeability was also reduced with the addition of 5 wt% of CNC to PVA/CMC films [119].

ZnO nanoparticles (ZnONPs) have been incorporated in a chitosan-based bionanocomposite and shown to be effective in the increment of the antimicrobial properties of the film, enhancing the intrinsic antimicrobial properties of the chitosan [95]. The incorporation of ZnONPs was also shown to be effective in the increment of the antioxidant properties, with the application of the produced films in fresh poultry meat decreasing its deterioration speed [95]. The effect of incorporating ZnONPs in a bilayer film for sponge cake packaging has been studied by Sahraee et al., 2020 [124]. A film composed of a first layer of N-chitin, ZnONPs, and gelatin, and a second layer of gelatin emulsion film, was demonstrated to be effective in maintaining the quality of the cakes, preventing fungal growth. The ZnONPs also improved the barrier properties of the film [124].

The mechanical and antimicrobial properties of polylactic acid (PLA) films with zinc oxide (ZnO) incorporated in three different concentrations (1, 3 and 5 wt%) have been evaluated [120]. The produced films were demonstrated to positively decrease CO_2_ and O_2_ permeability and mildly decrease water vapor permeability, and had good mechanical properties. The ZnO addition to the PLA films was shown to be positively effective as an antimicrobial agent against *E.coli* [120].

Swaroop and Shukla (2018) [121] used magnesium oxide nanoparticles (MgONPs) to reinforce PLA films for food packaging applications. These authors reported improvement in the mechanical and gas barrier properties in PLA films with MgONPs. In addition, the produced films showed good antibacterial properties. PLA-based nanocomposite films with the incorporation of zinc oxide nanoparticles have been studied [122], focusing on their effect on mechanical and water vapor properties, the UV-light barrier, and antibacterial properties. The addition of ZnO NPs did not significantly affect the UV-light barrier properties, showing just a slight decrease in the transparency of the film. The study demonstrated that ZnO NPs positively influenced the thickness, tensile strength, and water vapor barrier. When applied to minced fish paste, the films showed strong antibacterial properties against foodborne pathogens, extending the shelf life of the fish [122].

Starch films reinforced with MMT have been demonstrated to have the potential to be used in packaging for vegetables [110]. Starch/MMT films have also been demonstrated to increase the mechanical parameters of the films and no migrated MMT was found in spinach and lettuce after contact with the packaging material. The effect of MMT on rice flour-gelatin film with added catechin-lysozyme has been demonstrated to be effective in the prevention of lipid oxidation and microbial growth on fresh pork belly packaged with it [127]. The introduction of MMT and catechin-lysozyme increased the mechanical properties of film and its solubility in water but showed no effect on water vapor permeability; however, the nanoclay and antioxidant appeared to reduce the transparency and moisture content of film [127].

The addition of MMT and citric acid as reinforcement on whey protein isolate bionanocomposites has been studied by Azevedo et al., (2015) [79]. The interaction of MMT and citric acid led to a bionanocomposite with good potential features, e.g., mechanical, thermal, morphological, and structural properties, for application as food packaging material [79]. Bionanocomposites produced from potato peel wastes and with bacterial cellulose as a nano reinforcement agent reduced moisture content, water vapor, and oxygen permeability while improving the mechanical properties [123]. When curcumin was incorporated into the films, a positive effect on the preservation of fresh pork from lipid oxidation was observed [123].

Titanium dioxide (TiO_2_) has proved effective on the tensile strength and antioxidant activity increment of chitosan-starch films with clove essential oil addition, demonstrating its potential to be applied as food packaging material [125]. On the other hand, TiO_2_ incorporation leads to a decrease in water vapor permeability. Sago starch films reinforced with TiO_2_ and cinnamon essential oil (CEO) have been studied by Arezoo et al. (2019) [128]. The effect of TiO_2_ and CEO together led to improvement in the mechanical and barrier properties [128]; a similar reinforcement has also been reported on soluble soybean polysaccharide with TiO_2_ incorporation [129].

The addition of CNC and TiO_2_ (7.5 wt% and 0.6 wt%, respectively on wheat gluten-based films) has shown an improvement in water resistance and tensile strength [130]. TiO^2^ also exhibited good antimicrobial activity.

Nanocellulose is an incredibly versatile material that can be extracted from a variety of sources, including microorganisms, and shows great potential in food preservation, particularly when combined with bioactive compounds. For example, curcumin is a potent bioactive compound found in turmeric that has impressive antimicrobial properties [133]. By combining it with nanocellulose obtained from *Gluconacetobacter xylinus* bacteria, a composite material is formed that displays improved stability and biocompatibility [133]. Indeed, the work of Tangsatianpan et al. (2020) [133] has shown that this bionanocomposite has the ability to effectively inhibit the growth of harmful bacteria in food, making it a promising candidate for use as a food preservative. In other works, bacterial nanocellulose has been combined with propolis [134], a resinous substance produced by honeybees that has antimicrobial and antioxidant properties, or with nisin [135], a naturally occurring antimicrobial peptide that is effective against Gram-positive bacteria.

Multi-walled carbon nanotubes (MWCNTs) have been used to reinforce starch-based nanocomposites and found to be effective in improving mechanical properties [131]. The use of carbon nanotubes (CNT) and allyl isothiocyanate (AIT) for reinforcing cellulose-based films for sliced and cooked chicken meat packaging has demonstrated that CNT allows retention of the AIT on the films, thereby reducing microbial contamination, improving gas barrier properties, and retarding color changes [132].

#### 3.3.1. Examples of Bionanocomposites as Intelligent Packaging for Food Application

Bionanocomposites have applications in food packaging as an alternative to commonly used plastics [136]. Bionanocomposites can be used in the formulation of active and/or intelligent food packaging by detecting changes in the quality of the food product in real time, thus, providing this information to the consumer [13,137]. Bionanocomposites can be useful in monitoring food by sensing changes in temperature, pH, moisture, and gas composition, as well as detecting the presence of microorganisms through alteration of the packaging environment [138]. Moreover, the combination of bionanocomposites with bioactive compounds with antimicrobial and antioxidant features helps maintain product freshness and extend its shelf life [139]. Some examples of bionanocomposites that are incorporated with bioactive compounds to act as active and/or intelligent packaging are summarized in Table 3.

Most of the cases presented in Table 3 are freshness indicators that present a visual indication to the consumer. Freshness indicators can provide the entire food chain with information about the quality of a food product based on the production of metabolites that result in most cases from microbiological degradation [147]. The use of reinforcement agents in bionanocomposite production is crucial to improve several properties, such as mechanical, optical, thermal, and gas properties, that are extremely important in food packaging [139]. Furthermore, the addition of bioactive compounds to bionanocomposites improves the characteristics of a food packaging material by turning the package simultaneously into an active and intelligent system [148]. Thus, the use of bionanocomposites with bioactive compound addition emerges as a promising sustainable alternative for extending food shelf-life.

#### 3.3.2. Safety Aspects Related to the Use of Bionanocomposite as Food Contact Materials

Bionanocomposites are an emerging class of materials that combine biobased polymers with nanofillers to create materials with unique properties. These materials have attracted attention in recent years for their potential use as food contact materials due to their biodegradability, low toxicity, and renewability. However, before bionanocomposites can be used as food contact materials (FCM), their safety must be carefully evaluated to ensure that they do not pose a risk to human health [22,149,150].

One of the primary concerns regarding the use of bionanocomposites as FCM is the potential migration of NPs from the packaging material into the food; despite this, it is not fully established whether they can be released in nanosize form [22,151,152]. Nanoparticles have different properties and behavior than their larger counterparts, which may have implications for their safety [153,154]. Thus, the migration of NPs into food may result in unintended health effects for consumers [154].

To address these concerns, the European Food Safety Authority (EFSA) released in 2021 guidance on the risk assessment of nanomaterials to be applied in the food and feed chain: human and animal health [22]. The guidance includes the physicochemical properties, exposure assessment, and hazard characterization of nanomaterials and their determination in complex matrices. It also discusses the nano-specific considerations of toxicological studies and proposes a tiered framework for toxicological testing. The guidance outlines additional studies that may be needed to investigate potential risks, including reproductive and developmental toxicity, chronic toxicity and carcinogenicity, immunotoxicity and allergenicity, neurotoxicity, effects on gut microbiome, and endocrine activity. The EFSA proposes approaches to risk characterization and uncertainty analysis based on several relevant scientific studies, which will ensure the safe use of nanomaterials in food-related applications [22].

Overall, the results of these studies suggest that bionanocomposites have a good safety profile and can be used as food contact materials with appropriate precautions. According to Bott et al. (2017), if the nanoparticles in polymer nanocomposites are fully embedded within the polymer, and the contact surface remains unaltered by mechanical surface stress during application, it is likely that consumers will not be exposed to these NPs [155]. The migration of carbon nanotubes from low-density polyethylene (LDPE) and polystyrene (PS) was assessed in an early study by Bott el al. (2014), which proved that NPs do not migrate out of the matrix into food, as they are completely immobilized and their diffusion will always be smaller than the detection limit of any current sensitive method [156]. Moreover, the EFSA has released some scientific opinions on the safety of zinc oxide NPs [157] and modified montmorillonite clay [158], arguing that there are no safety concerns for the consumers exposed to such NPs if they are incorporated below some established limits. Moreover, in the case of ZnONPs, the EFSA also concluded that these particles do not migrate in the nanoform but are only soluble ionic zinc, and this should be the target of migration studies [159].

However, it is important to note that the safety of bionanocomposites may depend on several factors, including the type of biopolymer used, the type of nanofiller used, and the processing conditions [22,151]. Therefore, it is crucial to conduct safety evaluations on a case-by-case basis to ensure that the bionanocomposite material being used is safe for its intended use as a food contact material [95]. Continued research and evaluation of these materials will be necessary to ensure their safe use in food-related applications [155].

## 4. Conclusions and Outlook

The search for new and better food preservation techniques is an ongoing process that accompanies the success of the world’s largest food industries. Nanotechnology is highlighted as a promising tool for application in this important economic field to meet the need for novel preservation techniques. Its use through encapsulation with nanocarriers represents a promising approach for increasing the solubility and bioavailability of bioactive compounds. In this regard, the membrane emulsification process is expected to gain increasing importance, since it requires lower energy input, and the final droplet size of the emulsion can be tuned by a controlled pore size membrane selection. These are clear advantages for applications demanding stable emulsions with shear or heat-sensitive compounds. Another key advantage of the use of membrane emulsification is its easy scale-up ability.

As reinforcement material, nanotechnology can reduce the limitations of biodegradable packaging materials. Bionanocomposites can improve water vapor and gas barrier properties, thermal properties, mechanical properties, the UV barrier, biodegradability, and antibacterial and antifungal properties. The nanomaterials used to produce those bionanocomposites are mainly nanoclays, carbon nanotubes, nanocellulose, metal, and metal oxide nanoparticles with different particle sizes and shapes, and particular structural and functional properties. For the incorporation of nanofillers into the polymer matrix, different preparation methods may be used: intercalation, in situ polymerization, and melt processing. According to the dispersion of the nanofillers in the biopolymer matrix, three configurations can be obtained: intercalated (or partially exfoliated), exfoliated, or tactoid (micro-composite). The achievement of an exfoliation configuration is highly desired, as this results in the best improvement of the material’s mechanical and barrier properties.

When dealing with materials that will be consumed or come into direct contact with food that is intended for ingestion, it is crucial to establish regulations to ensure the safety of these products. Although some investigations have been conducted in this area, there is still a significant knowledge gap that needs to be addressed. Therefore, more research is needed, focusing on the potential biological effects that nanoparticles may have on the human body. This research should include tests for toxicity, migration, absorption, and digestion, among other factors. By conducting such research, we can better understand the potential risks associated with nanoparticles and develop regulations to ensure their safe use in food-related products.

## Figures and Tables

**Table 1:** Recent studies on bioactive compound encapsulation.

Nanocapsule—Wall Material	Bioactive Compound	Encapsulation Technique	Ref
Zein-Pectin	Resveratrol	Antisolvent precipitation and electrostatic deposition	[30]
Whey protein and chitosan	Garlic extract	Complex coacervation and spray-drying	[31]
Gelatin and sodium hexametaphosphate	Anchovy oil crosslinked with fucoidan extract	Complex coacervation and spray-drying	[32]
Lactoferrin-glycomacropeptide nanohydrogels	Nanohydrogels formed	Thermal gelation and nano spray-drying	[33]
Bovine lactoferrin	Omega-3 fatty acids	Nano spray-drying of the emulsion	[34]
Bovine serum albumin	Flavonoid rutin	Nano spray-drying	[35]
Stearic acid-chitosan conjugate and sodium caseinate	Astaxanthin	Ionic gelation and nano spray-drying	[36]
Gelatin	Ascorbic acid	Cryogelation	[37]
Sodium alginate and sodium carboxymethyl cellulose	*Eucalyptus camaldulensis* polyphenols	Freeze-drying	[38]
Gelatin-carrageenan	Gallic acid, catechin, chlorogenic acid, and tannic acid	Electric field-aided extrusion	[39]
Gelatin	Tomato peel carotenoids	Electro-spinning	[40]
Chitosan	Bromelain	Electro-spinning	[41]
Whey protein	Olive oil phenolic	Electro-spraying	[42]
Polyvinyl alcohol	Phycocyanin	Electro-spraying and electro-spinning	[43]
Oil with Tween 20 or Sodium dodecyl sulfate	Thyme essential oil	Emulsification	[44]
Cactus cladode mucilage	Zeaxanthin	Emulsification	[45]
Gelatin or whey protein	Melon carotenoids	Emulsification	[46]
Whey protein	Buriti and pequi carotenoids	Emulsification and freeze-drying	[47]
Chitosan	Clove essential oil	Emulsion and ionic gelation	[48]
Chitosan-Gum Arabic	Saffron Extract	Ionic Gelation	[49]

**Table 2:** Different types of bionanocomposites in food packaging.

Polymer	Nanoparticles (NPs)	Food Applied	Major Results	Ref
Gelatin/ginger essential oil	Montmorillonite (MMT)	-	MMT increases the thickness of the films. MMT decreases water solubility, moisture content, and superficial hydrophobicity.	[116]
Chitosan/rosemary essential oil	MMT	Fresh poultry meat	MMT improves barrier properties. MMT traps the phenolic compounds present in REO, avoiding its release into poultry meat.	[117]
Chitosan/papain	Cellulose nanofibers (CNF)	-	Papain impairs the dispersion of the CNF in the polymer matrix and increases crystallinity; however, the films show good transparency. CNF reduces the proteolytic activity of the films and retards the release of papain, due to changes in bionanocomposite crystallinity.	[118]
Polyvinyl alcohol/carboxymethyl cellulose (PVA/CMC)	Cellulose nanocrystals (CNC)	-	CNC decreases water vapor permeability. Tensile modulus and tensile strength increase with the addition of CNC to PVA/CMC films.	[119]
Chitosan	ZnO	Fresh poultry meat	Enhancement of the antimicrobial and antioxidant properties of the chitosan film. Films decrease the deterioration speed of fresh poultry meat.	[95]
Polylactic acid (PLA)	ZnO	-	ZnO addition increases mechanical properties. Antimicrobial activity against *E. coli.*	[120]
PLA	MgO	-	Good mechanical and gas barrier properties. Efficient antibacterial activity.	[121]
PLA	ZnO	Minced fish paste	Mechanical and water vapor improvement. Films applied in minced fish paste increase the shelf life of the product and have a strong antibacterial effect.	[122]
Potato peel/curcumin	Bacterial cellulose	Fresh pork	Bacterial cellulose is effective in the reduction of water vapor and oxygen permeability.	[123]
Starch	MMT	Lettuce and spinach	Improvement of mechanical properties. No migration to the vegetables.	[110]
Bovine gelatin/nano-chitin	ZnO	Cake	ZnO NPs improve barrier properties. Bilayer film prevents fungal growth on the cake.	[124]
Chitosan/starch/clove oil	TiO_2_	-	Improvement of tensile strength and antioxidant activity.	[125]
Chitosan/tocopherol	MMT	-	Incorporation of MMT improves the mechanical strength and thermal stability of the films. Incorporation of tocopherol provides antioxidant activity, improves the films’ thermal stability, and decreases tensile strength and elastic modulus, thus acting as a plasticizer.	[126]
Rice flour/gelatin/catechin-lysozyme	MMT	Fresh pork belly	Prevention of lipid oxidation and microbial growth. Increase in mechanical properties and film solubility.	[127]
Starch/cinnamon oil	TiO_2_	-	Mechanical and barrier properties improved.	[128]
Soluble soybean polysaccharide	TiO_2_	-	Mechanical and barrier properties improved.	[129]
Wheat gluten	TiO_2_ and CNC	-	Tensile strength and water resistance improvement. TiO_2_ exhibits good antimicrobial activity.	[130]
Whey protein/citric acid	MMT	-	Good mechanical, thermal, morphological, and structural properties.	[79]
Starch	Multi-walled carbon nanotubes	-	Mechanical properties improved.	[131]
Cellulose/allyl isothiocyanate	Carbon nanotubes (CNT)	Shredded and cooked chicken meat	Microbial contamination reduced. Improvement of the gas barrier.	[132]

**Table 3:** Bionanocomposites incorporated with bioactive compounds in food packaging.

Biomaterial	Bioactive Compound	Food Application	Function	Major Results	Ref
Gellan gum/Silver nanoparticles	-	Fresh chicken breast and silver carp	Intelligent packaging	The hydrogel is successfully produced to act as hydrogen sulfide (H_2_S) detector. When exposed to H_2_S, the hydrogel changes its color, providing information about the status of spoilage chicken and fish samples.	[140]
Chitosan/Graphitic carbon nitride	-	Tangerines	Active packaging	Films with chitosan and graphitic carbon nitride are successful. The addition of graphitic carbon nitride improves the mechanical, thermal, and barrier properties. When applied in tangerines (chitosan with graphitic carbon nitride (30%), antibacterial activity is produced, maintaining fruit freshness for 24 days.	[141]
Carboxymethyl cellulose/Cobalt-based metal-organic framework (Co-MOF)	-	Shrimp	Intelligent and active packaging	Films show promising results in terms of antibacterial and sensitivity to ammonia as well as color-stability to act as intelligent and active film. In addition, the application of shrimp and its freshness monitorization has been achieved.	[142]
Konjac glucomannan/Chitosan/Zinc oxide nanoparticles	Anthocyanins from Mulberry extract	-	Intelligent and active packaging	The addition of nanoparticles and anthocyanins to the film matrix improves mechanical, gas, thermal, and light-barrier properties. Moreover, films have been demonstrated to be pH-sensitive, by changing color according to the pH of the environment. Films also show good antioxidant and antibacterial properties.	[143]
Konjac glucomannan/Pullulan	Anthocyanins from Açai berry extract	Grass carps	Intelligent packaging	Pullulan has been used as a reinforcing agent to enhance the properties of films. The addition of açai berry extract to films improves several properties, namely water barrier, antioxidant, and antibacterial. In addition, films exhibit a color response when applied to fish, changing color according to degradation evolution due to pH alterations.	[144]
Carboxymethyl chitosan/Copper oxide nanoparticles	Anthocyanin from Saffron Petal	Lamb	Intelligent and Active packaging	The use of anthocyanins from saffron petals and nanoparticles enhances films in terms of their mechanical, thermal, antioxidant, and antibacterial properties. Moreover, anthocyanins also act as plasticizers. When in contact with lamb meat, films demonstrate freshness indicator ability.	[145]
Gelatin/Chitosan/Oxidized cellulose nanofiber	Curcumin and cinnamon oil	Pork	Intelligent and active packaging	Films have antioxidant features and increased UV-light barrier compared to pristine films. The use of bioactive compounds also allows a visual color change when pork degradation occurs.	[146]

## Data Availability

Not applicable.
